# Haematological cancer and quality of life: a systematic literature review

**DOI:** 10.1038/bcj.2015.29

**Published:** 2015-04-24

**Authors:** P Allart-Vorelli, B Porro, F Baguet, A Michel, F Cousson-Gélie

**Affiliations:** 1 Laboratory of Psychology ‘Health and Quality of Life' EA 4139, University Bordeaux Segalen, Bordeaux, France; 2 Department of Psychology, Epsylon EA 4556 Laboratory ‘Dynamics of Human Abilities and Health Behaviors', University Paul Valéry Montpellier 3, Montpellier, France; 3 ICM, Institut Régional du Cancer de Montpellier, Epidaure Prevention Unit - Rue des Apothicaires, Montpellier Cedex 5, France; 4 MIS, Montpellier Institut du Sein – 25 rue de Clémentville, Montpellier, France

## Abstract

The aim of this study is to examine the impact of haematological cancers on quality of life (QoL). A review of the international literature was conducted from the databases ‘PsycInfo' and 'Medline' using the keywords: 'haematological cancer', 'quality of life', 'physical', 'psychological', 'social', 'vocational', 'professional', 'economic', 'cognitive', and 'sexual'. Twenty-one reliable studies were analysed. Among these studies, 12 showed that haematological cancer altered overall QoL, 8 papers found a deterioration of physical dimension, 8 papers reported on functional and role dimensions, 11 papers reported on the psychological component and 9 on the social component. Moreover, one study and two manuscripts, respectively, reported deteriorated sexual and cognitive dimensions. Our review demonstrates that the different dimensions of QoL are deteriorated by haematological malignancies and, probably, by the side effects of treatment.

## Introduction

Haematological cancers include various diseases (Hodgkin's lymphoma, non-Hodgkin's lymphoma, leukaemia and multiple myeloma. The term leukaemia comprises acute myeloid leukaemia, chronic myelogenous leukaemia, acute lymphoblastic leukaemia and chronic lymphoblastic leukaemia); they can affect children, young adults and the elderly, and their incidence increases with age.^1^ As liquid tumours moving in the blood or lymph, acute or chronic diseases, with side effects induced by different treatments, are unique. Just as these diseases are distinct entities showing many differences from solid tumours, so too is the manner in which they are managed.^2^ In 1999, leukaemias and lymphomas accounted for approximately 8% of all cancers in adults.^3^ The 5-year survival rates vary from 47% to 95% depending on the malignancy.^4^

Quality of life (QoL) is usually impaired in the elderly: the biological nature and course of treatment of haematological cancer differ among children and adults,^5, 6, 7^ with long-term survival outcomes favouring young people diagnosed and treated as children.^3^

Our paper also focuses on health-related QoL (QoL), a factor reflecting the individual's assessment of his/her life at any one time relative to his/her previous state and prior experience.^8^ Health-related QoL is multidimensional and temporal, relating to a state of functional, physical, psychological and social/family well-being.^9^ Compared with the general population, the health-related QoL of cancer patients is worse in most dimensions.^10, 11^

This review describes the QoL and the different problems that patients with haematological malignancies encounter.

## Materials and methods

### Search strategy

A review was conducted from databases ‘PsycInfo' and ‘Medline', searching for studies published between 1990 and 2011 with keywords: ‘haematological cancer', ‘quality of life', ‘physical', ‘psychological', ‘social', ‘vocational', ‘professional', ‘economic', ‘cognitive', and ‘sexual' appearing in the abstracts.

We used nine combinations for all databases: (1) ‘QoL and haematological cancer', (2) ‘haematological cancer and physical', (3) ‘haematological cancer and psychological', (4) ‘haematological cancer and social', (5) ‘haematological cancer and cognitive', (6) ‘haematological cancer and economic', (7) ‘haematological cancer and professional', (8) ‘haematological cancer and vocational', and (9) ‘haematological cancer and sexual'.

#### Criteria for inclusion/exclusion

Prospective, comparative, exploratory, longitudinal or cross-sectional studies, assessing the QoL or health-related QoL, were analysed. Papers focusing on lymphoma, leukaemia or myeloma patients with chemotherapy, radiotherapy or blood transfusion in periods of remission or relapse were included. However, retrospective studies with other forms of cancer and reviews of the literature were excluded.

#### Quality assessment and levels of evidence

The studies had to be based on reliable methodological procedure (large population study, standardized tools and relevant statistical methods) and meet the criteria of a table that describes five levels of evidence (Level I: high-quality prospective study (all patients were enrolled at the same point in their disease with 80% follow-up of patients); Level II: retrospective study, untreated controls from a randomized control trial, lesser prospective study (patients enrolled at different points in their disease or <80% follow-up); Level III: case control study; Level IV: case series; Level V: expert opinion) in prognostic studies (investigating the effect of a patient's characteristic on the outcome of the disease).^12^ We considered only level I and II studies.

#### Data synthesis

Studies were analyzed by dimensions of QoL and symptoms (description of QoL in Table 1).

## Results

### Article identification

In total, 82 studies emerged: 73 studies for ‘PsycInfo' and 9 studies for ‘Medline'. There were 21 studies for combination 1, 14 for combination 2, 14 for combination 3, 9 for combination 4, 11 for combination 5, 3 for the combination 6, 10 for the combination 7 and 0 for the combinations 8 and 9. By limiting inclusion to studies that provided evidence of the impact of cancer on QoL, we selected 21 studies.

#### Methodological characteristics

One paper presented the level of evidence I^13^ and 20 level II.^14, 15, 16, 17, 18, 19, 20, 21, 22, 23, 24, 25, 26, 27, 28, 29, 30, 31, 32, 33^ Twelve were comparative,^14, 15, 16, 17, 18, 20, 23, 24, 26, 28, 30, 32, 34^ 5 were longitudinal,^14, 19, 20, 22, 33^ 5 were cross-sectional,^21, 23, 24, 26, 27^ 2 were descriptive,^16, 17^ 2 were pilot,^25, 29^ 1 was prospective,^23^ 1 was retrospective,^31^ 1 was international^28^ and 1 was a web-based survey.^28^

#### Patient population

In total, 7349 patients (3987 patients with lymphoma, 2303 with leukaemia, 711 with myeloma, 6 patients with amyloidosis and 1 with myelofibrosis; 341 no specified patients) were included in the studies (average age of 54.8 years).

One study focussed on the cognitive functioning of lymphoma patients by comparing two groups (test group and no-test group (diagnosis unknown).^15^ Another paper examined the QoL, without specifying the number of patients per diagnosis.^33^ The health-related QoL was studied in acute lymphoblastic leukaemia, myelofibrosis or unclassified leukaemia patients, but the authors did not specify the sample size of patients per diagnosis.^21^ There were 1171 control groups with haematological patients and healthy subjects in 3 studies.^20, 26, 32^

### QoL and health-related QoL of haematological cancer patients

#### Overall QoL

Twelve papers showed that haematological cancer negatively affect overall QoL and health-related QoL.^13, 14, 16, 18, 20, 22, 24, 25, 27, 28, 30, 31^ We noted a strong association between anaemia and QoL in lymphoma patients before chemotherapy.^22^ We found an impairment of QoL in multiple myeloma patients at diagnosis,^18^ at the beginning of treatment^13^ and during treatment,^27^ in chronic lymphoblastic leukaemia patients with chemotherapy^16^ and in multiple myeloma and acute lymphoblastic leukaemia patients at the start of chemotherapy.^25^ The latter study found that QoL was more deteriorated in patients with relapses, in comparison to patients who had no relapse, even at the onset of treatment. Moreover, QoL was worse in patients with an advanced stage of disease.^28^

Chronic lymphoblastic leukaemia patients had impaired health-related QoL compared with the general population.^16^ Compared with healthy controls, chronic lymphoblastic leukaemia patients with chemotherapy reported a lower QoL.^20^ Non-Hodgkin's lymphoma survivors with active disease presented a worse QoL compared with short- or long-term survivors.^30^ Moreover, one paper found a better QoL in Hodgkin's lymphoma survivors diagnosed 10–15 years previously than patients diagnosed 5–9 years ago.^24^

QoL improved after aerobic exercise training programme^14^ and was better in non-Hodgkin's lymphoma patients meeting public health exercise guidelines, compared with those who did not.^31^ Nevertheless, one study found that QoL of chronic lymphoblastic leukaemia patients was similar to or better than published population.^28^ However, one study demonstrated that QoL improved during and after chemotherapy in aggressive non-Hodgkin's lymphoma patients.^22^

#### General health

Five reports investigated the general health in haematological population.^14, 17, 22, 23, 24^ For multiple myeloma and non-Hodgkin's lymphoma patients, their physical health and mobility were the most frequent domains affected by the disease.^17^ Two studies noted that non-Hodgkin's lymphoma patients had a worse general health and that Hodgkin's lymphoma survivors presented lower general health compared with the population.^23, 24^ In another study comparing general health in patients treated with usual care or aerobic exercise training programme, aerobic exercise training patients had better general health than the other patients.^14^ However, after chemotherapy, general health improved in non-Hodgkin's lymphoma patients in one study.^22^

#### Physical dimension

Eight studies showed that haematological cancer deteriorates the physical component of QoL.^14, 18, 19, 20, 21, 24, 29, 30^ Some patients negatively perceived their physical well-being after bone marrow transplanation.^19^ Four other studies showed that physical function was affected in multiple myeloma patients^17, 18, 21, 29^ and that older patients presented more reduced physical functioning than younger patients.^21^ Non-Hodgkin's lymphoma survivors with active disease demonstrated worse physical functioning compared with disease-free survivors and population.^30^ Aerobic exercise training programme patients had better cardiovascular fitness than usual care patients.^14^ Long-term Hodgkin's lymphoma survivors diagnosed 10–15 years earlier reported better physical functioning than survivors diagnosed 5–9 years before.^24^ For chronic lymphoblastic leukaemia patients, physical functioning was significantly deteriorated compared with the healthy controls.^20^

Fatigue, lack of vitality and energy: Ten papers found that fatigue was one of the most prevalent symptoms experienced in haematological patients.^14, 15, 16, 18, 20, 21, 25, 26, 29, 32^ Compared with population, chronic lymphoblastic leukaemia patients had impaired health-related QoL for fatigue.^16^ For 55% of haematological patients, fatigue was the main symptom with insomnia,^21^ in particular in acute leukaemia and highly malignant lymphoma patients.^25^ One paper reported that levels of fatigue in Hodgkin's lymphoma and chronic lymphoblastic patients were higher than patients in healthy controls, even for years after treatment.^20^ Having severe illnesses in Hodgkin's lymphoma survivors was positively associated with fatigue.^26^ Another study showed that lymphoma patients who reported concentration and memory difficulties demonstrated much fatigue;^15^ symptom less pronounced in aerobic exercise training programme patients compared with usual care patients.^14^ These findings are consistent with the results found in another report.^18, 29^

Three studies noted that patients with bone marrow transplanation,^19^ Hodgkin's lymphoma survivors treated by radiotherapy or chemotherapy^32^ and Hodgkin's lymphoma survivors^24^ presented a lack of energy. For vitality, patients diagnosed 5–9 years before presented a greater lack of vitality than those diagnosed 10–15 years before.^24^ Finally, non-Hodgkin's lymphoma patients reported less vitality compared with population.^23^

Pain: Painful sensations were frequent in haematological patients for five studies.^18, 21, 22, 23, 29^ Pain was the most distressing problem for multiple myeloma,^18^ monoclonal gammopathy of unknown significance (MGUS)^29^ and leukaemia and lymphoma patients.^21^ In the latter study, older patients had more pain than younger patients. Similar results were found in non-Hodgkin's lymphoma patients who reported more bodily pain than the general population.^23^ However, during chemotherapy, less pain was experienced by aggressive non-Hodgkin's lymphoma patients in only one study.^22^

Sleep disorders: Four studies found that sleep was affected by haematological cancer.^16, 19, 22, 25^ Sleep disorders were prevalent in acute leukaemia and highly malignant lymphoma patients at the start of treatment.^25^ Compared with the general population, chronic lymphoblastic leukaemia patients presented more sleep disorders,^16^ related to functional well-being.^19^ An improvement was found in sleep disturbances during and after chemotherapy in aggressive non-Hodgkin's lymphoma patients.^22^

Digestive symptoms: Digestive symptoms may occur during haematological disease in four studies.^20, 21, 22, 33^ Among the most common problems in acute leukaemia patients, we found lack of appetite, weight loss, nausea and vomiting, 1 day after the end of chemotherapy or bone marrow transplanation. However, these symptoms had improved 10 and 21 days after the end of treatment.^33^ Older and recently hospitalised patients had more constipation, nausea and loss of appetite than younger patients and outpatients.^21^ Moreover, non-Hodgkin's lymphoma patients presented diarrhoea during chemotherapy but showed constipation 1 month after the end of treatment.^22^ Finally, chronic lymphoblastic leukaemia patients showed more nausea and vomiting, constipation and appetite loss than healthy controls.^20^

Dyspnoea: In three studies, dyspnoea, predominant with chemotherapy, was one of the most common symptoms in acute leukaemia and highly malignant lymphoma patients^25^ and in chronic lymphoblastic leukaemia patients.^16, 20^

Nutrition: In one study, nutritional deficits predominated in multiple myeloma and MGUS patients, treated with transplant.^29^

Fever: Only one study mentioned the problem of fever in acute leukaemia during chemotherapy.^33^

#### Functional and role dimensions

Two studies focussed on the functional dimension,^19, 28^ negatively affected after a bone marrow transplanation. However, in one study could most patients carry on with their daily activities without any help 1 year after bone marrow transplanation.^19^ Moreover, some authors found that daily functioning was similar or better than the population norms.^28^

Concerning role, six studies focussed on this dimension.^16, 18, 20, 21, 22, 25^ One study analysed the deterioration of role function in leukaemia, multiple myeloma or lymphoma patients.^21^ Role was affected in leukaemia and lymphoma^25^ and multiple myeloma patients,^18^ essentially in older patients. Compared with the general population, chronic lymphoblastic lymphoma patients had impaired role functioning for two studies.^16, 20^ However, improvement of role was observed 1 month after the end of chemotherapy in non-Hodgkin's lymphoma patients.^22^

#### Psychological dimension

Eleven studies showed that haematological cancers affect psychological QoL.^14, 15, 19, 20, 23, 24, 28, 29, 30, 32, 33^ One paper found that patients diagnosed 10–15 years earlier reported better psychological well-being than patients diagnosed 5–9 years ago.^23^ Lymphoma patients with chemotherapy presented possible anxiety and depression^15^ and we noted a high frequency of anxiety and depression in acute leukaemia patients, with a trend to improvement at the end of treatment.^33^ One study suggested that patients experienced more anxiety and mood disturbance after bone marrow transplanation compared with those with a longer follow-up.^19^ Hodgkin's lymphoma or non-Hodgkin's lymphoma patients receiving aerobic exercise training programme reported less depression and greater happiness compared with those who did not participate in the programme.^14^ Individuals with active disease demonstrated worse mental health functioning compared with population and disease-free survivors.^30^ Additional studies reported that emotional distress was present in multiple myeloma, MGUS, amyloid^29^ and chronic lymphoblastic leukaemia patients.^20^ Finally, chronic lymphoblastic leukaemia patients presented lower emotional well-being compared with the general population.^24, 28^ Moreover, Hodgkin's lymphoma survivors presented a different and positive vision of life after disease.^32^

#### Cognitive dimension

Two papers focussed on the cognitive functioning.^15, 20^ The cognitive area was significantly deteriorated in chronic lymphoblastic leukaemia patients, compared with healthy controls,^20^ and lymphoma patients with memory problems had a lower QoL.^15^

#### Social, professional and economic dimensions

Nine papers showed that social, professional and financial QoL were affected by haematological cancer.^17, 19, 20, 21, 23, 24, 25, 28, 32^ One report found a deterioration of social functioning in leukaemia and lymphoma patients with chemotherapy.^25^ Chronic lymphoblastic leukaemia patients presented a lower social QoL, mainly women.^20^ Another study found the same finding in patients diagnosed 5–9 years earlier compared with patients diagnosed 10–15 years before.^24^ In one study, the availability of, and satisfaction with, social support declined after bone marrow transplanation.^19^ The domain of family was affected in 89% of haematological patients.^17^ However, in one paper, the social functioning of chronic lymphoblastic leukaemia patients was similar to or better than that of the general population.^28^

Furthermore, one paper showed that Hodgkin's lymphoma survivors mentioned the topics of leisure and finance less frequently than controls.^32^ Older patients had fewer financial difficulties than outpatients, and multiple myeloma patients had a worse social QoL compared with those with other haematological cancers.^21^ Finally, most frequent domains mentioned were hobbies/pastimes, partnerships, profession and social life and friends^17^ and difficulties to obtain health-care insurance and life insurance.^23^

#### Sexual dimension

One study focussed on the sexual component^29^ and found that multiple myeoloma or MGUS patients presented sexual difficulties associated with body image.^29^ The problem of body image could be associated with hair loss mentioned in another paper.^33^

## Discussion

The general findings show that the haematological disease negatively affects overall QoL.^13, 14, 16, 18, 20, 22, 24, 25, 27, 28, 30, 31^ Compared with the general population, fatigue, pain or vitality were the more exposed^35, 36^ aspects of QoL, which were specifically deteriorated during an advanced stage of haematological cancer.^28^ Compared with the general population, haematological patients had an adverse general health.^23^ These results confirm other findings concerning cancer populations.^37^

Fatigue was the most prevalent physical symptom.^14, 15, 16, 18, 21, 25, 26, 29, 32, 33^ Most of the samples included elderly patients, and the progressive loss of autonomy in older people is not conducive to maintaining physical QoL. Haematological patients were more susceptible to fatigue than others because of the comorbidity and side effects due to treatment.^38^ Moreover, the benefits of physical programme on physical well-being were demonstrated.^27^ Similar data were found in Hodgkin's lymphoma survivors, an improvement in physical functioning and cardiovascular fitness being observed after exercise.^39^ The other physical symptoms were common to patients with other forms of cancers^40^ as well as breast cancer patients.^41, 42^

Only one study found that haematological patients can manage acts of daily life without the need for support after bone marrow transplanation.^19^ However, older and multiple myeloma patients experienced more reduced role function than younger patients and subjects with other diagnoses.^21^ Indeed, the multiple myeloma patients were older than patients with other diagnoses, and advanced age proved to be a predictor of symptoms. Role was more affected in haematological patients than in the general population.^16^ Because of physical disabilities, it is plausible that familial or social missions were disturbed.

Mostly, psychological QoL was found to be worse.^14, 15, 19, 20, 23, 24, 28, 29, 30, 32, 33^ One study noted that aerobic exercise training programme helped maintain good mental QoL.^27^ This may be due to the involvement of social interaction and a process of being distracted from one's cancer and treatments, a finding already made in advanced cancer patients.^43^ Moreover, emotional benefits occurred after patients with breast cancer followed a sports programme.^44^

Furthermore, haematological cancer damaged social, professional and financial QoL.^17, 19, 20, 21, 23, 24, 25, 28, 32^ Having cancer may improve social and familial relations by increasing the intensity of support and the availability of family caregivers. Conversely, emotional distress can affect the family sphere, and interpersonal relationships are likely to move towards the feeling of ambiguity or fear. Familial structure may be modified, leading to distress within the family. The deterioration of social well-being could be linked with self-image, including hair loss which increases after chemotherapy.^33^ Otherwise, the time since diagnosis may also have an impact on social QoL: in two studies, patients diagnosed 10–15 years earlier presented a better social QoL than those diagnosed 5–9 years before.^23, 24^ These findings were similar among families of patients with a head and neck cancer.^45^

Some studies found that professional life was negatively affected in patients.^23, 39, 41, 46^ Another study strengthened this finding by establishing that economic stress was negatively associated with QoL in gynaecological survivors.^47^ Moreover, one paper showed an increase in disability days in patients with breast, lung and gastrointestinal cancers.^48^ These consequences can lead to social isolation and frustration.^49^

Sexual activity, related to body image, was also reduced.^29^ Body image could be an important aspect of our criteria, with the fear of loss of masculinity or femininity and self-image. Other forms of cancer such as gynaecological malignancies also affected patients' sex life.^50^

With regard to cognitive functioning, haematological patients presented several memory and concentration disturbances.^28^ Similar results were found in cancer patients, in whom cognitive deficits were observed after chemotherapy.^51^

Physical, psychological, social and professional problems may be associated with the effects of treatment modalities. QoL was particularly affected in multiple myeloma and chronic lymphoblastic leukaemia patients, treated by chemotherapy or transplantation, in older patients, and in patients with active disease or an advanced stage of disease. Therefore, it would be interesting to conduct a further review with a synthesis of articles that highlight the impact on QoL of treatments recommended for a haematological malignancy.

The potential limitations of this review concern the literature search. Others involve the complexity in interpreting and measuring QoL, the heterogeneity of samples and the loss of subjects during research due to poor medical conditions, death or refusal.

## Conclusion

The major strength of our review is the reliability of the selected studies. It shows that haematological cancer patients have a poor QoL or health-related QoL compared with the general population. These findings hold regardless of the type of disease, the treatment modality and the stage of the disease. Generally, we found similar outcomes in other cancers, such as fatigue, which was greater in haematological patients. In theoretical terms, QoL is a complex concept that encompasses various aspects of life and is similar to well-being, so the very meaning of the notion is debatable. Clinically, it is important to analyse QoL early in the course of care. Some types of intervention may prove helpful such as physical programmes, which may be considered as a form of functional care intervention, and other supportive actions, such as psychotherapy which can improve physical and mental functioning.

## Figures and Tables

**Table 1:** Impact of haematological cancer on QoL or HRQoL

*Article*	*Design*	*Population*	*Disease status/treatment*	*Procedure*	*Psychometrics instruments*	*Results*
Cull *et al.*^15^	Comparative	Test group: 91 patients: - 55 HL - 36 NHL No-test group:^a^ 109 patients^b^	Disease-free Chemotherapy	Patients completed the instruments and returned them with a form giving preferred times for objective testing	QLQ-C30 HADS MFI BMFQ Memory aids NART PASAT RBMT	Poor QoL in complainers of memory problems No-test group reported better cognitive functioning but more fatigue than the test group (*U*=2.0, *P*=0.05) Test group: 30% patients reported difficulty in concentrating, 52% in remembering things, 63%, memory impairment, 13% possible anxiety and 10% possible depression
Zittoun *et al.*^33^	Longitudinal	179 acute leukaemia patients	Chemotherapy or BMT	T1: 1 day after the end of chemotherapy or BMT T2: 10 days later the end of chemotherapy T3: 21 days after the end of chemotherapy	QLQ-C30 HADS Leukaemia/BMT module	Lack of appetite (T1=64%, T2=53%, T3=49% *P*=0.03), fever (T1=24%, T2=47%, T3=22% *P*⩽0.001), nausea (T1=38%, T2=23%, T3=16% *P*⩽0.001), vomiting (T1=35%, T2=17%, T3=16% *P*⩽0.001), and hair loss (T1=19%, T2=54%, T3=60% *P*⩽0.001) High frequency of anxiety and depression over time Trend to improvement at the end of treatment
Heinonen *et al.*^19^	Longitudinal	109 patients: - 32 CML - 39 AML - 15 ALL - 13 MDS - 5 MM - 2 NHL - 2 AA - 1 myelofibrosis	Allogeneic BMT	T1: ⩽12 months after BMT T2: 12 to ⩽36 months after BMT T3: 36 to ⩽60 months after BMT T4: >60 months after BMT	FACT-BMT POMS ADL Scale MOS-SS SSQ6	T1: worse perception of physical well-being (*P*=0.000, PV=20%), anxiety (⩽12: MS=9.8; s.d.=5.0; over 12: MS=7.6; s.d.=4.2; *F*= 4.1*) and mood disturbance (⩽12: MS=49.1; s.d.=25.0; *F*=4.8*) Deterioration of availability and satisfaction with social support Most of patients could carry on with their daily activities without any help Functional well-being negatively affected (T1=16.8%, T2=27.1%, T3=21.5%, T4=34.6%) because of lack of energy and sleeping disorders
Persson *et al.*^25^	Longitudinal	16 patients: - 7 AL - 9 HML	Chemotherapy 7 patients had no relapse 6 patients had a relapse 3 patients died before remission	T1: start of treatment T2: 4 months after treatment T3: 8 months T4: 12 months T5: 16 months T6: 20 months T7: 24 months	QLQ-C30	T1: Deterioration of QoL, role and social functioning (more important in AL than HML patients) with fatigue, dyspnoea and sleep disturbances Deterioration of QoL in patients with relapses than those without relapse T7: Deterioration of role, social, cognitive and emotional functioning and poor QoL, essentially in patients with relapses Deterioration of role and social functioning more important in AL than HML patients
Wettergren *et al.*^32^	Comparative	357 subjects: - 121 HL survivors - 236 CG	Long-term survivors Radiation, chemotherapy or combined modality	Scales sent by postal mail Respondents were promised a movie ticket if they participated in the study	SEIQoL-DW	HL survivors reported leisure and finance less frequently than CG (leisure: HL survivors=31.4% and CG=47.9% df=1; *P*=0.003; finance: HL survivors=29.8% and CG=41.5% df=1; *P*=0.03) Fatigue, loss of energy in HL patients (10.7% *N*=13 on 121; MS=3.0; s.d.=1.0)
Sherman *et al.*^29^	Pilot	61 patients: - 52 MM - 5 MGUS - 4 amyloid	Patients newly admitted to the transplant programme Stage of diseases:^c^ - 13 for the stage I - 10 for the stage II - 32 for the stage III - 6 unknown	Assessment prior to starting local protocols for conditioning and transplant	SF-12 POMS-F PG-SGA BPI HADS FACIT	Major symptoms: nutritional deficits, deterioration of physical functioning, fatigue and pain, emotional distress, disrupted sexual functioning and difficulties with body image
Rüffer *et al.*^26^	Comparative	1753 subjects: - 818 HL survivors - 935 CG	Long-term survivors Radiotherapy, chemotherapy Combined modality treatment	In 1995, the authors had contacted 1981 patients, who were enrolled in the German Hodgkin Studies Patients with a current status of complete remission were contacted to participate in this study	QLQ-S QLQ-C30 LSQ MFI	All levels of fatigue are high even years after treatment and higher than those of the CG: GF: Patients: MS=37.6; s.d.=29.1/CG: MS=30.9; s.d.=23.2; *P*<0.001 PF: Patients: MS=32.6; s.d.=29.2/CG: MS=25.0; s.d.=24.2; *P*<0.001 RA: Patients: MS=28.0; s.d.=26.1/CG: MS=21.4; s.d.=21.8; *P*<0.001 MF: Patients: MS=26.6; s.d.=26.8/CG: MS=21.8; s.d.=23.5; *P*<0.001 RM: Patients: MS=19.8; s.d.=20.1/CG: MS=16.9; s.d.=18.1*; P*<0.001 Association between severe illnesses with fatigue
Gulbrandsen *et al.*^18^	Comparative	Data from two prospective Nordic Myeloma Study Group Trials: - 221 patients <60 years treated with high-dose chemotherapy - 203 patients >60 years treated with MP	Patients newly diagnosed with MM, with addition to low-dose IFN alpha 2b to conventional treatment with MP Comparison of results with the Norwegian population	Data obtained from prospective trials	QLQ-C30	At diagnosis, most distressing problems: pain, fatigue, reduced physical functioning, limitations in role functioning and reduced QoL
Frick *et al.*^17^	Comparative	46 MM 20 NHL 13 other diseases^d^	Complete remission: 6 patients Partial remission: 52 patients No change: 15 patients Progressive disease: 1 patient Not available PBSCT: 5 patients	Randomisation: - individualised Psychodynamic short term Psychotherapy immediately after PBSCT until 6 months after PBSCT - from 6 months after PBSCT until 1 year after - to a CG receiving ‘treatment as usual'	QLQ-C30 SEIQoL-DW	Most frequent domains nominated by the patients: family (89%), hobbies/pastimes (74%), physical health (mobility) (70%), profession/occupation (51%), social life/friends (47%) and partnership (33%)
Merli *et al.*^22^	Longitudinal	91 aggressive NHL	Chemotherapy	T1: diagnosis and before treatment T2: during chemotherapy T3: 1 month after the end of chemotherapy	QLQ-C30	T1: association between anaemia and poor QoL T2: improvements of QoL, pain (*P*=0.003), appetite (*P*=0.006), sleep (*P*=0.015) and GH (*P*=0.027), except diarrhoea and social activity T3: improvements of QoL (*P*=0.05), global health (*P*=0.011), appetite (*P*=0.0001), emotional functioning (*P*=0.01) and role (*P*=0.05), reductions of pain (*P*=0.02), sleep disorders (*P*=0.007) and constipation (*P*=0.04)
Holzner *et al.*^20^	Longitudinal	76 CLL 152 HC	Chemotherapy	T1: baseline T2: 3 months after baseline T3: 6 months after baseline T4: 12 months after baseline	QLQ-C30	Lower QoL in CLL patients compared with HC Physical functioning: effect size medium (ES −0.56; *P*<0.001) Role and cognitive functioning: effect size small (ES −0.43, *P*<0.01 and ES −0.27, *P*<0.1) More symptoms in CLL patients compared with HC: fatigue (ES 0.81), nausea and vomiting (0.69), constipation (0.69), appetite loss (0.68) and dyspnoea (0.44) Lower emotional and social QoL in female than in male patients
Vallance *et al.*^31^	Retrospective	438 NHL survivors: - 255 indolent - 183 agressive	- 283 with chemotherapy - 68 with chemotherapy and radiation - 47 with radiation - 16 with surgery - 4 with immunotherapy - 36 with BMT	Questionnaire mailed to patients	FACT-An	Better QoL in NHL meeting public health exercise guidelines than NHL not meeting guidelines
Santos *et al.*^27^	Cross-sectional	107 patients: - 54 NHL - 18 AML - 10 ALL - 25 MM	In treatment: - 42 with intravenous chemotherapy - 5 with oral medication - 5 with radiotherapy - 55 with monitoring	Instruments applied during face-to-face interviews	QLQ-C30	Deterioration of QoL essentially in MM patients, contrary to patients with other cancers
Mols *et al.*^24^	Comparative	116 long-term HL survivors^e^ - 48 patients for the 5–9 year survivors - 68 patients for the 10–15 year survivors	Off-treatment	Survey conducted at the ECR^f^	SF 36 QoL-CS	Better QoL in patients diagnosed 10–15 years ago compared with patients 5–9 years ago Lower GH and lack of energy in patients diagnosed 10–15 years ago than patients diagnosed 5–9 years ago Lower general and mental health, social functioning and vitality in patients diagnosed 5–9 years ago, compared with normative sample
Mols *et al.*^23^	Comparative	221 NHL Sample size for population is not specified	Long-term survivors (5–15 years postdiagnosis)	Recruited from the ECR	SF-36 QoL-CS	Patients diagnosed from 10 to 15 years earlier reported better psychological (*β*= 0.17*) and social (*β*= 0.21**) well-being than patients diagnosed from 5 to 9 years earlier Compared with population, patients reported worse GH (*P*<0.001), less vitality (*P*<0.001), higher scores for pain (*P*<0.001) and problems with work or obtaining health-care insurance and home mortgage
Shanafelt *et al.*^28^	Comparative	1482 CLL	Majority of patients with low-stage disease at diagnosis 40.3% of patients with chemotherapy and/or monoclonal antibody	Between June and October 2006	FACT-G BFI	QoL and social and functional dimensions were similar to or better than population norms QoL was worse in patients with advanced stage of disease Lower emotional well-being in CLL patients, compared with population and patients with other types of cancer
Else *et al.*^16^	Comparative	431 CLL	Chemotherapy	Randomisation into the Leukaemia Research Fund CLL4 trial Instruments given at the start of chemotherapy	QLQ-C30	Impaired HRQoL for the fatigue, sleep disturbance, role functioning and dyspnoea in CLL patients compared with population
Strasser-Weippl and Ludwig^13^	Randomized clinical trial	92 MM	Chemotherapy	Questionnaires presented to patients during the first study visit of the clinical trial Conventional treatment	QLQ-C30	Impairment of QoL at onset on therapy
Courneya *et al.*^14^	Longitudinal	122 patients: - 52 indolent NHL - 48 aggressive NHL - 22 HL	62 patients with UC 60 patients with AET	T0: baseline T1: postintervention T2: 6-month follow-up	FACT-An Happiness Scale Depression Short Form Center for Epidemiological Studies-Depression Scale STAI SF-12	T1: better physical functioning (mean group difference, +9.0; CI=2.0 to 16.0; *P*=0.012), cardiovascular fitness (*P*<0.001), QoL (*P*=0.021), happiness (*P*=0.004) and GH (*P*<0.001) and attenuation of fatigue (*P*=0.013) and depression (*P*=0.005) in AET compared with UC patients T2: better happiness (*P*=0.034), and attenuation of depression (*P*=0.009) in AET, comparatively UC patients
Johnsen *et al.*^21^	Cross- sectional	470 patients: - 34 AML - 132 CLL - 34 CML - 33 HL - 164 NHL - 54 MM - 19 ALL, myelofibrosis or unclassified leukaemia	- 269 patients had lymphoma stage 1 or 2 - 60 patients had inaccessible lymphoma stage - 99 patients had no treatment - 358 patients had active antineoplastic treatment	Scales, information letter and consent form send by mail	QLQ-C30	Symptoms experienced by patients: fatigue (55%), insomnia (46%) and pain (37%) Impairments: role (49%) and physical functions (34%) More problems (physical, role, social functions, pain and constipation) in MM in comparison to other patients More reduced in physical (OR^2^=1.53; 95% CL= 1.36–1.74; *P*<0.001) and role functions (OR^*2*^=1.32; 95% CL= 1.16–1.51; *P*<0.001), constipation (OR^2^=1.47; 95% CL=1.22–1.78; *P*<0.001), appetite loss (OR^2^=1.28; 95% CL=1.08–1.51; *P*=0.004) and pain (OR^2^=1.28; 95% CL=1.13–1.45; *P*<0.001) in older patients, compared with younger patients, but less financial difficulties (OR^2^=0.76; 95% CL=0.65–0.89; *P*<0.001) in older patients More nausea (OR^2^=2.98; 95% CL=1.30–6.83; *P*=0.010) and appetite loss (OR^2^=3.14; 95% CL=1.33–7.41; *P*=0.009) in recently hospitalised patients than outpatients
Smith *et al.*^30^	Comparative	761 NHL survivors: - 109 patients with ‘active disease' - 150 ‘short-term survivors' - 502 ‘long-term survivors'	STS (2–4 years postdiagnosis) LTS (⩾5 years postdiagnosis)	Scales send by mail	SF-36 FACT-G FACT-LYM IOC Self-administered Comorbidity Questionnaire PTSD Checklist	Lower QoL, physical (mean (s.d.), 41.1 (11.9)) and mental health (mean (s.d.), 45.4 (11.5)) (all *P*⩽0.01) in individuals with active disease, compared with survivors

Abbreviations: AA, acute amyloid; ADL, Activities of Daily Living Scale (Katz *et al.*, 1970); AET, aerobic exercise training; AL, acute leukaemia; ALL, acute lymphoblastic leukaemia; AML, acute myeloid leukaemia; BFI, Brief Fatigue Inventory (Mendoza *et al.*, 1999); BMFQ, Brief Mental Fatigue Questionnaire (Bentall *et al.*, 1993); BMT, bone marrow transplantation; BPI, Brief Pain Inventory (Cleeland, 1989); CG, control group; CLL, chronic lymphocytic leukaemia; CML, chronic myelogenous leukaemia; ECR, Eindhoven Cancer Registry; FACT-An, Functional Assessment of Cancer Therapy—Anaemia (Cella, 1997); FACT-BMT, Functional Assessment of Cancer Therapy—Bone Marrow Transplant (McQuellon *et al.*, 1197); FACT-G, Functional Assessment of Cancer Therapy-General (Cella *et al.*, 2003); FACIT, Functional Assessment of Chronic Illness Therapy (Cella, 1997); GF, general fatigue; GH, general health; HADS, Hospital Anxiety and Depression Scale (Snaith et Zigmond, 1983); HRQoL, health-related quality of life; HL, Hodgkin's lymphoma; HML, highly malignant lymphoma; IFN, interferon; IOC, Impact Of Cancer (Zebrack *et al.*, 2008); LSQ, Life Situation Questionnaire (Joly *et al.*, 1996); LTS, long-term survivors; MA, mean age; MCS, Mental Component Summary; MDS, myelodysplastic syndrome; MF, mental fatigue; MFI-20, Multidimensional Fatigue Inventory (Smets *et al.*, 2000); MM, multiple myeloma; MGUS, monoclonal gammopathy of unknown significance; MIRT, Myeloma Institute for Research and Therapy; MOS SS, Medical Outcome Study Social Support (Sherbourne & Stewart, 1991); MP, Melphalan and Prednisone; NART, National Adult Reading Test (Nelson, 1991); NHL, Non-Hodgkin's Lymphoma; PASAT, Paced Auditory Serial Addition Task (Gronwall & Sampson, 1974); PF, Physical Fatigue; PBSCT, peripheral blood stem cell transplantation; PCS, Physical Component Summary; PG-SGA, Patient-Generated Subjective Global Assessment (Ottery, 1996); POMS, Profile of Mood States (McNair *et al.*, 1971); POMS-F, Profile of Mood States—Fatigue Scale (McNair *et al.*, 1992); PTSD Checklist, PostTraumatic Stress Disorder Checklist (Weathers *et al.*, 1993); QLQC30, Quality of Life Questionnaire C30 (Aaronson *et al.*, 1983); QLQ-S, Quality of Life Questionnaire—Survivors (Sprangers *et al.*, 1993); QoL, quality of life; QoL-CS, quality of life—cancer survivors; RA, reduced activity; RBMT, Rivermead Behavioural Memory Test (Wislon *et al.*, 1991); RM, reduced motivation; SEIQoL-DW, Schedule for the Evaluation of the Individual Quality of Life-Direct Weighting (Browne *et al.*, 1997); SF-12, Short Form 12 items (Ware, 1995); SF-36, Short-Form 36 items; SSQ6, Brief Measure of Social Support (Sarason *et al.*, 1987); STAI, Spielberger State Anxiety Inventory (Spielberger, 1993); STS, short-term survivors; UC, usual care. *P<0.05; **P<0.01.

a Patients who did not present for psychometric testing with respect to age, sex, employment, educational status, diagnosis, therapy received or duration of treatment.

b MA not specified in the manuscript.

c Staged using Durie and Salmon classification.

d MA not specified, most patients are between 50 and 60 years.

e MA not specified, most patients are between 35 and 49 years.

f This register identifies all patients newly diagnosed with cancer in the southern part of the Netherlands.
